# Laparoscopic Proctocolectomy With Transanal Total Mesorectal Excision for Ulcerative Colitis

**DOI:** 10.7759/cureus.19720

**Published:** 2021-11-18

**Authors:** Yuya Ashitomi, Hiroshi Oshio, Mitsuhiro Yano, Shinji Okazaki, Fuyuhiko Motoi

**Affiliations:** 1 First Department of Surgery, Yamagata University, Faculty of Medicine, Yamagata, JPN; 2 Department of Surgery, Nihonkai General Hospital, Yamagata, JPN

**Keywords:** laparoscopic surgery, proctocolectomy, p53, ulcerative colitis, transanal total mesorectal excision

## Abstract

Transanal total mesorectal excision (TaTME) refers to endoscopic retrograde total mesorectal excision and is becoming increasingly popular worldwide. TaTME improves surgical manipulation and minimizes the risk of local recurrence of rectal cancer by ensuring circumferential resection margins. TaTME is mainly indicated for patients in whom transabdominal approaches are expected to be technically challenging. We extended the indications for TaTME to include surgery for ulcerative colitis lesions that might be cancerous in the rectum. Here, we report a case of proctocolectomy with TaTME for ulcerative colitis. A 38-year-old woman who was receiving treatment for ulcerative colitis underwent a biopsy for random samples from the transverse colon to the rectum. Histopathological findings revealed noninvasive dysplasia with p53 overexpression, suggestive of cancer. We extended the indication of TaTME to surgery for ulcerative colitis. We formed two surgical teams and performed laparoscopic proctocolectomy with TaTME simultaneously. This simultaneous operation reduced the duration of the procedures in the present case. The patient was discharged without any complications and underwent loop ileostomy closure four months postoperatively. The patient recovered without significant loss of the anal sphincter function and is doing well four months after the second surgery. We propose laparoscopic proctocolectomy with TaTME to be conducted simultaneously by two teams as a safe and effective technique that is associated with a shorter operation time than that reported previously. Additionally, TaTME was useful in confirming the appropriate dissection layer as well as in surgical manipulation. Hence, TaTME could serve as a useful therapeutic option for ulcerative colitis surgery.

## Introduction

Transanal total mesorectal excision (TaTME) refers to endoscopic retrograde total mesorectal excision (TME). This is mainly considered for patients in whom transabdominal approaches are expected to be technically challenging, such as in patients with extensive adhesions, giant tumors, and lower rectal cancers, particularly in a narrow male pelvis [1,2]. Proctocolectomy is a highly invasive procedure that requires extensive manipulation [3]. Minimally invasive procedures are preferred, particularly in patients with noninvasive cancer. Laparoscopy is a minimally invasive approach that provides better cosmetic outcomes than open surgery, although the operation time tends to be longer. It has been reported that proctocolectomy with concomitant mucosectomy may cause postoperative anal dysfunction [4]. Here, we report a case of TaTME combined with proctocolectomy for ulcerative colitis lesions that might be cancerous in the rectum. We formed two surgical teams that performed the operation simultaneously to ensure safety, reduce the operation time, improve fine surgical dexterity, and achieve stable optical magnification for mucosectomy.

## Case presentation

A 38-year-old woman presented with a 16-year history of ulcerative colitis being treated with mesalazine and infliximab. She had been undergoing routine colonoscopy examinations. Multiple random biopsies performed from the transverse colon to the rectum during colonoscopy suggested rectal cancer, for which she was referred to our hospital for surgery. A follow-up colonoscopy revealed the absence of the haustra between the transverse colon and rectum, without any obvious tumors. Histopathological examination of multiple random biopsy specimens obtained from the transverse colon to the rectum showed dysplasia with p53 overexpression in the rectum, which suggested cancer.

**Figure 1:**
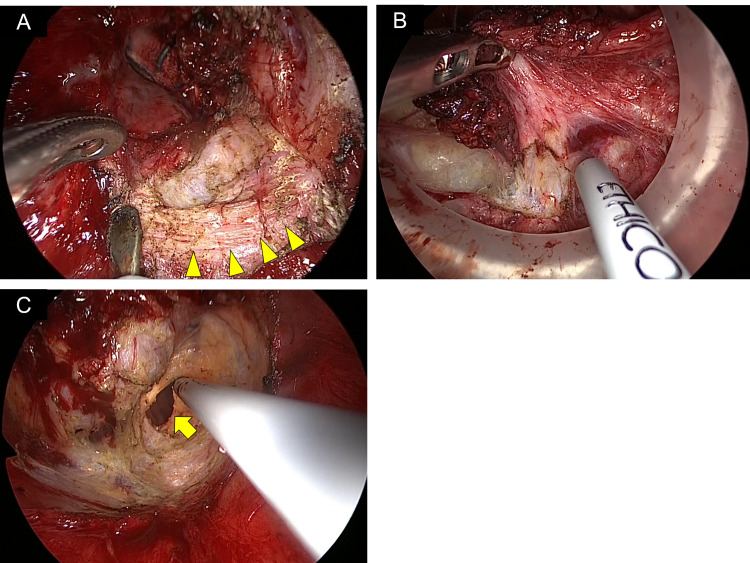
Intraoperative findings in a patient who underwent transanal total mesorectal excision. (A) Circumferential mucosectomy preserves the internal sphincter muscle (arrowhead). (B) Dissection of the hiatal ligament. (C) Image showing the opened abdominal cavity (arrow).

We performed laparoscopic proctocolectomy and D2 lymphadenectomy concomitantly with TaTME. The procedure involved two surgical teams. We inserted five abdominal ports, and the colon was mobilized from the ileocecal region to the rectum along with laparoscopy-guided dissection of blood vessels. The rectum was mobilized in the TME plane, and the left and right neurovascular bundles were incised. Transanal surgery was performed simultaneously using the laparoscopic procedure. We used the Lone Star Retractor System (Cooper Surgical, Trumbull, CT, USA), GelPOINT path transanal access platform (Applied Medical, Rancho Santa Margarita, CA, USA), and AirSeal system (ConMed, Utica, NY, USA) to ensure active smoke evacuation to aid in the visualization of the operative field. Circumferential mucosectomy was performed with preservation of the anal sphincter muscle. We used a purse-string suture and closed the rectal lumen to prevent mucus leakage and cancer cell dissemination. Mucosectomy was performed starting from the dentate line and extending into the anal canal. The circular and longitudinal muscles were incised, and the abdominal cavity was opened (Figure 1). The specimen was extracted after a slight extension of the umbilical port site. We created an ileal pouch (J-pouch) and performed an ileal pouch-anal anastomosis. Finally, we performed a diverting-loop ileostomy. The operation time was 286 minutes, and the estimated blood loss was 52 mL.

Histopathological examination of the resected specimen revealed low-grade rectal dysplasia without any evidence of malignancy. It took some time to adjust to the ostomy pouch, and the patient was discharged 21 days postoperatively without any complications. The patient underwent loop ileostomy closure four months postoperatively and recovered without significant loss of the anal sphincter function. The anal sphincter function remained at four months after the second surgery. At four months after the second surgery, the patient is doing well.

## Discussion

TME is important to prevent recurrence and the need for rectal surgery [5]. Surgery that ensures a circumferential resection margin (CRM) can prevent local recurrence; however, a CRM is often difficult to achieve and depends on the site and size of the tumor [6]. TaTME is a surgical approach that uses transanal laparoscopic devices to perform a retrograde TME. Lacy et al. introduced this procedure and referred to it as a “down-to-up TME” [7]. TaTME improves surgical manipulation and minimizes the risk of local recurrence by ensuring a CRM using TME. This is particularly of value for patients in whom transabdominal surgery is technically challenging, such as in patients with extensive adhesions, giant tumors, a narrow pelvis, and lower rectal cancers [1,2,8]. The TaTME Registry Collaborative database reported outcomes of 2,653 consecutive patients who underwent TaTME. It was observed that the rate of positive CRM was 4.0% in the study [9].

A few reports have described laparoscopic proctocolectomy using TaTMEs. Leo et al. reported 16 cases of proctocolectomy performed concomitantly with TaTME for ulcerative colitis and concluded that TaTME obviated the repeated use of staplers and facilitated the safe dissection of the lower rectum [10]. Ambe et al. reported eight cases of proctocolectomy with TaTME for familial adenomatous polyposis and observed no perioperative complications [11]. Therefore, these authors recommend TaTME as an effective and safe treatment strategy.

Recently, robot-assisted surgery has been introduced for total colorectal resection [12,13]. The safety of robot-assisted surgery for total colorectal resection is not inferior to that of laparotomy or laparoscopy. However, there are issues regarding surgical technique and insurance coverage, and further studies are needed in this regard.

Table 1 shows a comparison between the findings of the present case and those observed in five previously reported cases of conventional laparoscopic proctocolectomy.

**Table 1:** Comparison of the clinical data of the present case with those of five patients who underwent conventional laparoscopic proctocolectomy. ASA: American Society of Anesthesiologists

Case	Age	Sex	ASA score	Operation time (minutes)	Complications	Hospital stay (days)
1	59	F	2	368	Enteritis	23
2	27	M	2	263	None	11
3	19	F	2	328	Ileus	56
4	68	M	3	317	None	28
5	29	F	2	424	Abscess formation	23
Present case	38	F	2	286	None	21

The mean operation time was 340 minutes in the previous cases mentioned in Table 1. Currently, at our hospital, TaTME is indicated for difficult surgical cases of advanced rectal cancer after preoperative chemotherapy and for male intersphincteric resection cases [14]. However, we have now expanded the indication for TaTME to include ulcerative colitis lesions that might be cancerous. The operation was performed by two surgical teams in the present case; one team performed the laparoscopic procedure, and the other team performed the transanal procedure. Simultaneous operations reduced the operation time in the present case. The present case required more careful surgical manipulation because of the possibility of cancer. Although deep pelvic manipulation is prone to an inadequate field of vision, the two teams were able to perform the operation while mutually confirming the appropriate dissection layer. In addition, the magnifying effect provided by the endoscope during the TaTME improved tissue visualization as well as maneuverability, which minimized thermal nerve injury and improved postoperative anorectal function. Moreover, no postoperative complications were observed, and the postoperative hospital stay was comparable to that reported in previous cases.

However, the following are some of the disadvantages of TaTME: (1) accurate understanding of the anatomical structures and training in surgical techniques are essential. The TaTME Registry Collaborative reported that an incorrect dissection plane was observed in 7.8% of all cases and visceral injury was observed in 1.5% of all cases [15]. (2) Surgeons who are not proficient in performing TaTME-specific techniques may increase the local recurrence rate of rectal cancer, as discussed in a previous study from Norway [16]. While TaTME is considered useful, a drawback is that only a limited number of cases are indicated for TaTME. We believe that this problem may be solved by expanding the indications for TaTME to include other benign diseases, such as an anal stricture. (3) This approach requires at least five surgeons (two teams). Despite these disadvantages, TaTME may serve as a useful technique for laparoscopic proctocolectomy.

## Conclusions

We reported a case of laparoscopic proctocolectomy with TaTME for ulcerative colitis lesions that could have been cancerous. In the case of this patient, two surgical teams successfully performed the proctocolectomy, suggesting the usefulness of the TaTME approach. TaTME facilitates mucosectomy and improves functional disorders after ulcerative colitis surgery. Thus, it could serve as a useful therapeutic option for ulcerative colitis surgery. Finally, the usefulness of TaTME for benign diseases such as ulcerative colitis needs to be verified by future clinical studies.

## References

[1] Narihiro S, Ohdaira H, Takeuchi H (2020). Transanal total mesorectal excision (Ta-TME) in a rectal cancer patient with a history of abdominal surgery: a case report. J Anus Rectum Colon.

[2] Veltcamp Helbach M, Koedam TW, Knol JJ, Velthuis S, Bonjer HJ, Tuynman JB, Sietses C (2019). Quality of life after rectal cancer surgery: differences between laparoscopic and transanal total mesorectal excision. Surg Endosc.

[3] Boller AM, Larson DW (2007). Laparoscopic restorative proctocolectomy for ulcerative colitis. J Gastrointest Surg.

[4] Lindquist K (1990). Anal manometry with microtransducer technique before and after restorative proctocolectomy. Sphincter function and clinical correlations. Dis Colon Rectum.

[5] Heald RJ, Husband EM, Ryall RD (1982). The mesorectum in rectal cancer surgery--the clue to pelvic recurrence?. Br J Surg.

[6] Quirke P, Durdey P, Dixon MF, Williams NS (1986). Local recurrence of rectal adenocarcinoma due to inadequate surgical resection. Histopathological study of lateral tumour spread and surgical excision. Lancet.

[7] de Lacy AM, Rattner DW, Adelsdorfer C (2013). Transanal natural orifice transluminal endoscopic surgery (NOTES) rectal resection: "down-to-up" total mesorectal excision (TME)--short-term outcomes in the first 20 cases. Surg Endosc.

[8] Fukase M, Oshio H, Murai S (2019). Transanal total mesorectal excision of giant villous tumor of the lower rectum with McKittrick-Wheelock syndrome: a case report of a novel surgical approach. Surg Case Rep.

[9] Roodbeen SX, de Lacy FB, van Dieren S (2019). Predictive factors and risk model for positive circumferential resection margin rate after transanal total mesorectal excision in 2653 patients with rectal cancer. Ann Surg.

[10] Leo CA, Samaranayake S, Perry-Woodford ZL (2016). Initial experience of restorative proctocolectomy for ulcerative colitis by transanal total mesorectal rectal excision and single-incision abdominal laparoscopic surgery. Colorectal Dis.

[11] Ambe PC, Zirngibl H, Möslein G (2017). Initial experience with taTME in patients undergoing laparoscopic restorative proctocolectomy for familial adenomatous polyposis. Tech Coloproctol.

[12] Flynn J, Larach JT, Kong JC, Warrier SK, Heriot A (2021). Robotic versus laparoscopic ileal pouch-anal anastomosis (IPAA): a systematic review and meta-analysis. Int J Colorectal Dis.

[13] Kim JC, Lee JL, Yoon YS (2020). Entirely robot-assisted total colectomy/total proctocolectomy compared with a laparoscopic approach. Surg Laparosc Endosc Percutan Tech.

[14] Oshio H, Oshima Y, Yunome G (2021). Transanal total mesorectal excision and transabdominal robotic surgery for rectal cancer: a retrospective study. Ann Med Surg (Lond).

[15] Penna M, Hompes R, Arnold S (2017). Transanal total mesorectal excision: International Registry results of the first 720 cases. Ann Surg.

[16] Larsen SG, Pfeffer F, Kørner H (2019). Norwegian moratorium on transanal total mesorectal excision. Br J Surg.

